# Effect of Botulinum Toxin Injection on the Progression of Hip Dislocation in Patients with Spastic Cerebral Palsy: A Pilot Study

**DOI:** 10.3390/toxins13120872

**Published:** 2021-12-06

**Authors:** Yookyung Lee, Seungeun Lee, Joonyoung Jang, Jiwoon Lim, Ju Seok Ryu

**Affiliations:** 1 Department of Rehabilitation Medicine, Seoul National University Hospital, Seoul National University College of Medicine, Seoul 03080, Korea; yookon@gmail.com; 2 Department of Rehabilitation Medicine, Seoul National University Bundang Hospital, Seoul National University College of Medicine, Seongnam-si 13620, Korea; tmddms5843@nate.com (S.L.); drjyjang@gmail.com (J.J.); jlovej71@gmail.com (J.L.)

**Keywords:** cerebral palsy, hip dislocation, electromyography, botulinum toxin, pathophysiology

## Abstract

Hip adductor spasticity is a contributing factor to hip dislocation in patients with cerebral palsy (CP). We hypothesized that botulinum toxin injected into the hip adductor muscles would reduce spasticity and help prevent hip dislocation. Twenty patients with bilateral spastic CP aged 2 to 10 years with gross motor function classification system level IV or V were included. Botulinum toxin was injected into the hip adductor muscles at baseline and at 6-month follow-up. Muscle tone was measured with an eight-channel surface electromyography (EMG) recorder. A hip X-ray was performed, and Reimer’s hip migration index (MI) was measured. The Wilcoxon signed-rank test was used to compare the surface EMG values of the hip muscles at baseline and follow-up. The mean root mean square surface EMG value of the hip adductor muscles was significantly reduced at 1, 2, 3, and 7 months after the first injection, up to approximately 53% of the baseline. The 1-year progression of the hip MI was −0.04%. Repeated sessions of botulinum toxin injections at the hip adductor muscles significantly reduced muscle tone and hip displacement. A botulinum toxin injection may be used as an adjunctive treatment in the prevention of hip dislocation.

## 1. Introduction

Factors associated with hip dislocation in patients with spastic cerebral palsy (CP) can be divided into three categories, namely, developmental, protective, and mechanical. Developmental factors include weight bearing and ambulation [1]. In non-ambulatory patients with CP, the lack of physiologic mechanical stress on the hip joint leads to coxa valga, femoral anteversion, and acetabular dysplasia [1,2], which increase the risk of hip dislocation. Protective factors are muscles and ligaments. Mechanical factors are hip adductor muscle spasticity and an imbalance in hip muscle tone. Increased adduction forces on the hip joint are thought to create torque on the femoral head, shifting it laterally out of the acetabulum [3,4,5,6].

A botulinum toxin injection is a widely used treatment for hip adductor spasticity [7]. However, its effectiveness in preventing hip dislocation remains unclear [7,8]. Several studies have reported that a botulinum toxin type A injection into the hip adductor muscles results in stable or improved hip displacement [9,10,11,12]. Other studies reported that a botulinum toxin A injection does not significantly improve hip displacement [13,14,15]. Previous findings are inconsistent with regard to the effectiveness of a botulinum toxin injection into the hip adductor muscles in improving hip displacement.

Multiple factors are associated with hip dislocation; thus, no single treatment may be effective in preventing hip dislocation. However, we hypothesized that a botulinum toxin injection would produce positive effects with regard to the prevention of hip dislocation by causing neurogenic denervation and atrophy of the muscle [16]. We hypothesized that such atrophic changes would lead to a lasting reduction in hip adductor spasticity, an important contributing factor to hip dislocation. We also hypothesized that the reduction in hip adductor spasticity would subsequently decrease hip displacement. Therefore, the aim of this study was to quantitatively evaluate the trajectory of hip adductor muscle spasticity after repeated sessions of botulinum toxin injections and assess its effect on the progression of hip dislocation.

## 2. Results

Patient characteristics are presented in Table 1. Twenty patients (fourteen males and six females) were included in the study. The mean age of the patients was 5 years and 1 month (with a standard deviation of 1 year and 10 months). Three and seventeen patients were classified with gross motor function classification system (GMFCS) levels IV and V, respectively.

**Figure 1:**
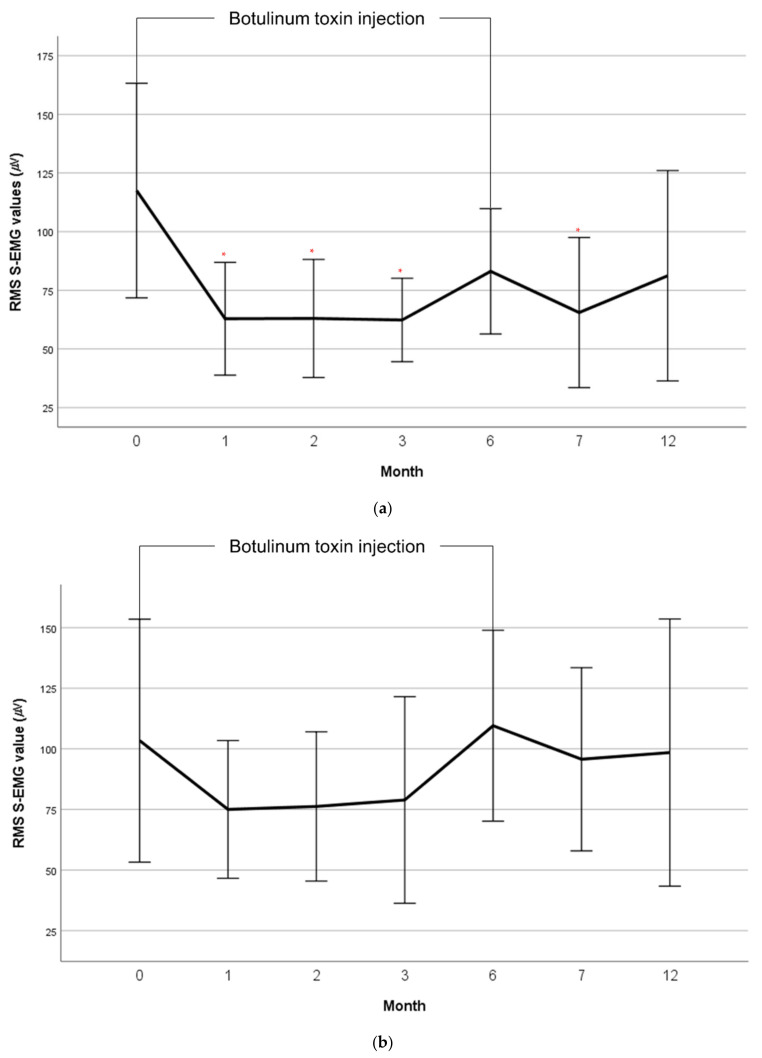
Mean RMS S-EMG value changes of the hip adductor muscles (**a**) and abductor muscles (**b**) by month. RMS, Root Mean Square; S-EMG, Surface electromyography; Error bar: Standard error. * *p* value < 0.05.

The mean root mean square (RMS) surface electromyography (EMG) values of the hip adductor and abductor muscles by month are shown in Table 2. The Friedman test showed no significant differences in the RMS values between the baseline and follow-up. Post hoc analysis of Wilcoxon signed-rank test was performed. The mean RMS surface EMG values of the sum of the adductor muscles were significantly reduced compared with the baseline at 1, 2, 3, and 7 months (Figure 1a). At 3 months, the mean RMS surface EMG values of the adductors were reduced to approximately 53% of the baseline. The mean RMS surface EMG value at 12 months remained at 70% of the baseline, however it was not statistically significant. The mean RMS surface EMG values of the abductor muscles showed a similar activation pattern to those of the adductor muscles (Figure 1b).

The progression of the hip migration index (MI) in the study patients after one year was −0.04% (8.64). This was lower than 3.27% (8.62) of the historical control data of patients with CP recruited at the same center, although this is not statistically significant (Table 3). The hip abduction range of motion (ROM) with a 90° hip flexion had significantly improved from 29.5 (16.54) to 45 (0) by the 12-month follow-up visit. Hip adductor modified Ashworth scale (MAS) grade was significantly reduced at 12 months compared with the baseline. Satisfaction and quality of life after a botulinum toxin injection, measured using the Caregiver Priorities and Child Health Index of Life with Disabilities (CPCHILD), significantly improved when compared with the baseline (Table 4).

## 3. Discussion

A botulinum toxin injection into the hip adductor muscles, followed by repeat injection at 6 months, significantly reduced muscle tone at 1, 2, 3, and 7 months after the first injection. Hip adductor muscle tone remained below baseline levels at 12 months, although this was not statistically significant. The progression of the hip MI per year was significantly lower than that of the control group.

Previous studies using MRI evaluations have shown that a botulinum toxin injection causes neurogenic muscle atrophy due to chemo-denervation of the muscle [17,18,19]. The reason that the hip adductor muscle tone did not return to baseline levels at 6- and 12-month follow-up may be attributed to neurogenic atrophy of the muscles. Muscle atrophy is usually considered to be an adverse effect of the botulinum toxin injection. However, in non-ambulatory patients with CP, hip adductor muscle tone has little functional value. Neurogenic muscle atrophy may even be desirable considering its role in reducing spasticity.

Our study showed that hip adductor muscle activation was reduced after a botulinum toxin injection. Since spasticity is velocity dependent, our surface EMG recordings were measurements of involuntary background muscle activation and not spasticity [20]. However, both velocity dependent and non-velocity dependent muscle activation are important mechanical factors in hip dislocation. Both spasticity and non-velocity dependent muscle activation share a common pathway of increased muscle tone. Therefore, we considered reduced muscle activation as reflection of concomitant reduction in spasticity.

The hip abductor muscle tone showed a similar activation pattern to the hip adductor muscle tone (Figure 1b). Several explanations seem possible. The hip abductor muscles are antagonists to the hip adductor muscles. Therefore, a similar activation pattern of the abductor muscles can be explained by co-contraction [21]. Reduced hip abductor muscle tone reflects reduced hip adductor tone after a botulinum toxin injection. Another possible explanation is that the similar activation reflects dystonic features. Spasticity and dystonia frequently coexist, and abnormal movement frequency has been reported to increase with higher GMFCS levels [22]. However, a reduction in hip abductor muscle tone after a botulinum toxin injection into the hip adductor muscles cannot be fully explained by dystonic features.

In a previous study by Terjesen [23], the hip MI progression per year was 3.9% for patients with CP classified as GMFCS level IV and 9.5% for patients classified as GMFCS level V. The hip MI progression per year was 3.27% in our control group, which consisted of 24 patients with CP classified as GMFCS levels IV and V. The slightly lower than expected hip MI progression in our control group may be attributed to the inclusion of both hips in the analysis. Terjesen [23] only included the side with the largest hip migration percentage. We included both hips as the bilateral hip adductors were spastic and a botulinum toxin was injected into both sides. In the botulinum toxin injection group, the hip MI progression rate per year was −0.04%, which was lower than the control group, although not statistically significant. Our study results were comparable with those from a previous study by Yang et al. [9], who compared hip displacement between the botulinum toxin injection group and the surgery group. In low functioning patients (GMFCS levels III, IV, and V), the annual changes in hip MI were 0% and −1.0% for the botulinum toxin injection group and the surgery group, respectively [9].

Two studies examined the effect of a botulinum toxin injection, along with abduction bracing on hip dislocation [13,14]. Both studies reported minimal or no significant effect as a result of a botulinum toxin injection on the prevention of hip dislocation. The negative results may be attributed to the inclusion of patients with a relatively lower risk of hip dislocation (patients classified as GMFCS levels I-III). In our study, we included only patients with CP classified as GMFCS levels IV and V, patients with high risk of hip dislocation. In addition, abduction bracing may have masked the effect of a botulinum toxin injection. Recent studies have reported that abduction bracing can aggravate hip displacement due to leverage effects and length tension relationship [4,5]. A recent retrospective cohort study by Lin et al. [15] also reported no significant differences in hip dislocation and hip surgery rate in the botulinum toxin injection group and the control group. However, it was a retrospective study; thus, detailed information on the GMFCS levels and the protocols of the botulinum toxin injection administration was lacking. A significantly lower mortality was observed in the botulinum toxin injection group, however the reason for this was unclear [15]. Multiple factors are associated with hip dislocation, and the results of previous studies suggest that a botulinum toxin injection alone may not be sufficient to prevent hip dislocation. Our study results showed that a botulinum toxin injection is effective in reducing hip adductor spasticity, an important factor in hip dislocation, and patients with CP may have additional benefits from the botulinum toxin injection.

The botulinum toxin injection significantly improved quality of life, as measured using the CPCHILD. In a previous study, the mean CPCHILD scores of children classified as GMFCS levels IV and V were 46.0 (20.1) and 24.3 (18.6), respectively [24]. The CPCHILD score in our study was 25.44 (17.39) at baseline, and it significantly increased to 33.59 (17.64) at 1 month. This remained in the range of 38 to 39 throughout the study period. A previous study also reported a significantly improved CPCHILD score at 16 weeks after a botulinum toxin injection in patients with CP [25]. A botulinum toxin injection can improve the quality of life and ease of care by reducing spasticity. The quality of life and satisfaction measured using the Likert questionnaire were the highest at 1 month after a botulinum toxin injection and the lowest at 12 months.

Our study has several limitations. First, this was a pilot study. Our study was not a confirmatory trial with randomization and calculation of sample size. Correlation between reduced hip adductor spasticity and decreased hip displacement could not be confirmed. Second, 6 patients did not complete the 12-month follow-up and some patients with severe hip displacement were lost in follow-up or received hip surgery. There is a risk of selection bias. However, we still think a botulinum toxin injection may have a role in delaying the progression of hip displacement by reducing hip adductor spasticity, a mechanical factor contributing to hip dislocation, regardless of hip displacement severity. What is unclear is whether patients with severe hip displacement will benefit from a botulinum toxin injection or not, and further studies are needed to determine the answer. Third, the follow-up period was 12 months. The long-term effect of a botulinum toxin injection on hip dislocation was not assessed. Alternatively, we measured the change in Reimer’s hip MI over 1 year as a measure of hip displacement progression. We also quantified the change in hip adductor muscle tone, an important contributing factor to hip dislocation, over time. Fourth, the hip adductor muscle tone was not directly measured. Muscle activation was indirectly measured using the surface EMG. Some discrepancies may exist between actual muscle tone and the surface EMG measurements. The strength of this study was the quantitative measurement of muscle tone. Qualitative evaluation of spasticity using MAS has poor reliability in patients with CP [26]. Fifth, surface EMG values were not normalized but expressed with the RMS method. This method does not control for differences between sessions such as electrode placement and subcutaneous tissues [27]. However normalization methods, such as maximal voluntary contraction, are not clinically possible in children due to problems in cooperation. Sixth, other factors which may affect spasticity such as medication and physical therapy were not controlled. However, these factors were not considered significant since the candidates for a botulinum toxin injection were patients who had spasticity despite medication or physical therapy. Lastly, the GMFCS level of the children was imbalanced, with only 3 children with GMFCS level IV and 17 with GMFCS level V. Caution is needed in generalizing the results. However, GMFCS level is a significant risk factor for hip dislocation [28]. Our study reported results in the patients with the highest risk.

## 4. Conclusions

In conclusion, a botulinum toxin injection is effective in reducing hip adductor muscle tone and spasticity. Hip adductor spasticity is an important contributing biomechanical factor to hip dislocation, and a botulinum toxin injection may be used in conjunction with other treatments in order to prevent hip dislocation. Future studies with a larger sample size and a longer follow-up period are needed to confirm the effect of a botulinum toxin injection in improving hip dislocation.

## 5. Materials and Methods

Patients with spastic CP were included in this prospective observational study conducted between December 2018 and July 2020. They received a botulinum toxin injection into the hip adductor muscles and a repeat injection after 6 months. The patients were followed-up for 1 year. This study was approved by the Institutional Review Board of our hospital (Approval no.: B-1807-478-001). Legal guardians of all the patients provided written informed consent to the research and to the publication of the study results before participation. Patients were recruited from two hospitals.

### 5.1. Participants

To date, no studies have published the changes in surface EMG values after a botulinum toxin injection into the hip adductor muscles. Since this is the first clinical trial, we included 20 consecutive patients in the study. Inclusion criteria were a diagnosis of bilateral spastic CP [29], aged 2 to 10 years, and GMFCS level IV or V. Exclusion criteria were as follows: contraindication to botulinum toxin injection (such as hypersensitive reactions to botulinum toxin), history of a botulinum toxin injection within 6 months prior to study enrollment or scheduled for injection at another institution after study enrollment, history of hip surgery or scheduled for hip surgery after study enrollment, and on drugs that interact with botulinum toxin (such as aminoglycoside, spectinomycin, polymyxin, tetracycline, lincomycin, and tubocurarine muscle relaxant).

### 5.2. Procedures

**Figure 2:**
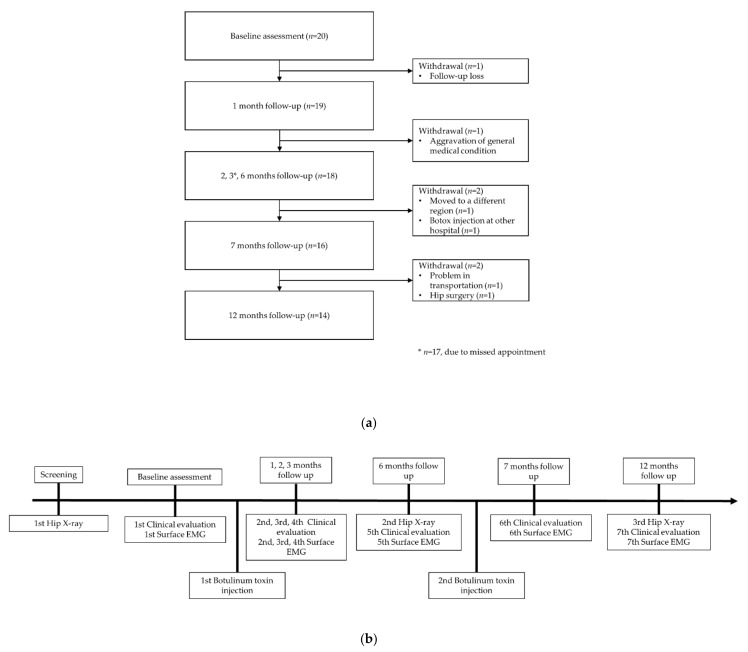
(**a**) Patient follow-up and (**b**) study flow.

Patient demographic data and medical history were collected by the researchers during the screening process. Eligible patients underwent height and weight measurements and initial hip radiographic evaluation. Clinical evaluation and recording of the first hip muscle surface EMG were performed at the initial visit. After the first botulinum toxin injection, the patients were followed up at 1, 2, 3, and 6 months. Following a repeat injection at 6 months, the patients were followed-up at 7 and 12 months after the first botulinum toxin injection (Figure 2a). At each visit, clinical evaluation and surface EMG recording of the hip adductor and abductor muscles were performed. Patients also underwent hip radiographic evaluation at 6- and 12-month follow-up visits. To assess satisfaction levels and the quality of life after a botulinum toxin injection, the Likert questionnaire and the CPCHILD were administered at each visit (Figure 2b). Twenty patients were enrolled, and fourteen patients completed the 1-year follow-up (Figure 2a).

The injection site of the muscle was initially checked with an ultrasound to mark the injection location. Topical lidocaine cream (EMLA cream 5%) was applied on the muscles prior to the injection. Botulinum toxin was then injected at the motor point under EMG guidance. Ultrasound and EMG-guided injection could not be administered concomitantly due to a lack of cooperation from children. Clostridium botulinum toxin type A (Meditoxin^®^, 200 units) product was diluted with 4 mL of normal saline to produce a dilution of 50 units/1 mL. Botulinum toxin was injected into the bilateral adductor longus and adductor magnus muscles at a dose of 3 U/kg and into the bilateral adductor brevis and gracilis muscles at a dose of 1 U/kg for a total maximum dose of 16 U/kg of body weight or a maximum dose of 200 units overall. The distribution of the botulinum toxin injection over the different muscles was based on muscle size. For patients receiving 200 units the botulinum toxin dosage was fixed at 100 units at for each side, 12.5 units at the adductor brevis and gracilis muscles, and 37.5 units at the adductor longus and magnus muscles. Injection was repeated at 6 months.

### 5.3. Outcomes

Clinical evaluation included hip and knee ROM and MAS of hip adductor muscle spasticity. Hip and knee ROM was measured using a goniometer. Hip abduction ROM was measured with the hip and knee in 90° of flexion, and with the hip and knee in neutral position. Knee flexion ROM was measured at the supine position with the hip fully flexed. Knee extension ROM was also measured at the supine position with the hip flexed to 90° (Popliteal angle test) [30]. The MAS is an ordinal measure of spasticity scored on a 0–4 scale, with a score of 1+ between 1 and 2 [26]. A score of 0 denotes no increase in muscle tone, and a score of 4 indicates the affected part was rigid in flexion or extension. Hip adductor spasticity was measured with the knee flexed. Quality of life, comfort, ease of care, and overall health were evaluated with Likert scale questionnaire. A score of 5 indicates very satisfied, and a score of 1 denotes very unsatisfied. The Korean version of the CPCHILD was also administered. The CPCHILD is a measure of caregivers’ perspectives on child health, function, and well-being, and has six domains scored on a 0–100 scale and in total. For each of the six domains, standardized scores from 0 (worst) to 100 (best) were calculated as well as for the total survey [24].

Hip abductor and adductor muscle tone evaluation was conducted with a wireless S-EMG analysis system (BTS FREEEMG 1000 with EMG-BTS EMG-Analyzer; BTS Bioengineering Co., Garbagnate Milanese, Italy) using electrophysiological quantitative analysis. Surface EMG electrodes were placed on the muscle belly of the bilateral adductor longus, adductor magnus, tensor fascia latae, and gluteus medius muscles. With the patients in the seated position, involuntary background muscle activation was provoked by slow stretch movement. The peak amplitude and RMS values were recorded for 5 s and measured for 1 s at the plateau. Surface EMG RMS values were measured twice, and the mean value was used. Since measurements were taken from both sides, the values were averaged to represent the muscle tone. The surface EMG values of the adductor longus and adductor magnus muscles were added to produce the sum of the adductor muscles. Similarly, the surface EMG values of the tensor fascia lata and gluteus medius muscles were added to produce the sum of the abductor muscles. Surface EMG analysis was performed at baseline and 1, 2, 3, 6, 7, and 12 months.

Plain radiographs of the hip in the supine position were taken at baseline and at 6- and 12-month follow-up visits. The Reimer’s MI was digitally measured using the Picture Archiving and Communication System (Impax, Agfa, Antwerp, Belgium) by two assessors blind to the timing of the X-ray. Inter-observer correlation coefficient was computed and the mean value was used for analysis. We used the change in Reimer’s MI over 1 year as a measure of the progression of hip displacement. The MI of both sides was included.

### 5.4. Statistical Analysis

Statistical analyses were performed using SPSS 25.0 software (SPSS Inc., Chicago, IL, USA). The baseline hip adductor and abductor muscle tone were compared with the hip adductor and abductor muscle tone at 1-, 2-, 3-, 6-, 7-, and 12-month follow-up visits by using the Friedman test and the Wilcoxon signed-rank test. Hip and knee ROM, hip adductor spasticity, and CPCHILD scores at follow-up were also compared with those at baseline using the Wilcoxon signed-rank test. For analysis, hip adductor spasticity MAS grade of 0 was coded as 0, grade of 1 as 1, grade of 1+ as 2, grade of 2 as 3, grade of 3 as 4, and grade of 4 as 5. The Mann–Whitney U test was used to compare the progression of the hip MI over 1 year with that of the historical control data from a previous study [4], consisting of 24 patients with cerebral palsy, aged 2 to 10 years, and classified as GMFCS level IV or V (Table 1). The patients were being followed-up every 6 to 9 months at our center according to the hip surveillance guidelines [31,32,33]. A *p* value < 0.05 was considered significant. Missing data was addressed by performing complete case analysis.

## Figures and Tables

**Table 1:** Demographics and clinical characteristics.

Variables	Intervention (*n* = 20)	Control * (*n* = 24)	*p*-Value **
Age (yr)	5.1 (1.86)	6.13 (2.47)	0.13
Age group (yr)			
2–4	9	7	
4–6	5	5	
6–8	6	8	
8–10	0	4	
Sex (M:F)	14:6	13:11	0.29
GMFCS level (IV:V)	3:17	11:13	<0.01
Height (cm)	100.6 (12.27)		
Weight (kg)	15.07 (4.41)		
Hip Migration index			
Right	38.19 (22.53)	30.70 (18.94)	0.25
Left	39.14 (29.26)	30.14 (14.77)	0.22
Hip and Knee ROM			
Hip abduction (with hip 90’ flexion)	29.5 (16.54)		
Hip abduction (with hip extension)	24.5 (15.64)		
Knee flexion	111 (63.73)		
Knee extension (Popliteal angle)	9.75 (14.19)		
Hip Adductor spasticity (MAS)			
<2	9		
≥2	11		
Orthoses			
Spinal orthoses	0		
Postural support	3		
Ankle foot orthoses	8		
Anti-spasticity medication	7		

Data are presented as mean (standard deviation). GMFCS, Gross Motor Function Classification System; ROM, Range of Motion; MAS, Modified Ashworth Scale. * Historical control data of patients with CP recruited at the same center. ** Independent-samples *t*-test for parametric, Mann– Whitney U test for nonparametric variables.

**Table 2:** Mean RMS S-EMG values (μV) of the hip adductor, abductor muscles by month.

	Baseline	1 Month	2 Months	3 Months	6 Months	7 Months	12 Months
Adductor longus m.	61.62 (56.27)	30.03 (24.18) *	34.14 (32.76)	32.79 (18.49)	43.75 (35.20)	42.18 (46.41)	45.40 (51.67)
Adductor magnus m.	55.90 (49.52)	32.84 (34.65) *	28.86 (23.28) *	29.55 (20.36) *	39.34 (23.56)	23.32 (19.92) *	35.80 (34.36)
Adductor muscles sum	117.52 (102.28)	62.87 (52.37) *	63.00 (53.37) *	62.35 (36.67) *	83.09 (56.63)	65.50 (63.97) *	81.20 (83.87)
Tensor fascia lata m.	78.55 (96.32)	47.63 (39.77)	50.21 (44.82)	53.68 (63.52)	72.64 (69.99)	61.53 (50.36)	69.25 (83.94)
Gluteus medius m.	24.84 (24.37)	27.37 (29.83)	26.01 (32.20)	25.21 (27.08)	36.90 (26.89)	34.17 (33.95)	29.21 (27.05)
Abductor muscles sum	103.39 (112.10)	75.00 (61.93)	76.22 (65.32)	78.89 (87.86)	109.54 (83.55)	95.69 (75.56)	98.46 (103.12)

Values are expressed in as mean (standard deviation). RMS, Root Mean Square; S-EMG, Surface electromyography. * *p* value < 0.05.

**Table 3:** One year progression of the hip migration index (%) compared to control.

	Botox Injection (*n* = 14)	ICC ^1^	Control ^2^ (*n* = 24)
Hip migration index			
Baseline	31.84 (16.23)	0.98	30.43 (16.85)
6 months	31.95 (13.69)	0.97	
12 months	31.80 (14.27)	0.95	
One year progression rate	−0.04 (8.63)		3.27 (8.62)

Values are expressed as mean (standard deviation). ^1^ Historical control data of patients with CP recruited at the same center. ^2^ ICC, Inter-observer correlation coefficient.

**Table 4:** Changes of Hip and knee ROM, Hip adductor spasticity (MAS), CPCHILD and Likert Questionnaire after botulinum toxin injection.

	Baseline	1 Month	2 Months	3 Months	6 Months	7 Months	12 Months
ROM							
Hip abduction (with hip 90’ flexion)	29.50 (16.54)	37.37 (12.4)	36.94 (14.87)	41.56 (7.90) *	41.76 (7.06) *	43.67 (3.52) *	45.00 (0.00) *
Hip abduction (with hip extension)	24.50 (15.64)	37.37 (12.84) *	34.17 (14.68) *	40.31 (9.03) *	30.29 (9.43)	42.33 (5.63) *	37.69 (10.33)
Knee flexion	111.00 (63.73)	135.79 (42.86)	133.89 (46.92)	144.06 (23.75)	141.18 (36.38)	150.00 (0.00)	142.31 (27.74)
Knee extension (Popliteal angle)	9.75 (14.19)	0.79 (2.5) *	0.83 (3.54) *	7.50 (21.21)	3.24 (10.15)	0 (0) *	2.31 (8.32)
Hip Adductor spasticity (MAS)							
<2 ≥2	911	136	135	143	153	142	122
CPCHILD	25.44 (17.39)	33.59 (17.64) *	39.30 (19.94) *	38.93 (20.14) *	39.73 (22.00) *	39.22 (17.56) *	39.29 (13.62) *
Questionnaire (Likert scale)							
Quality of life		3.89 (0.81)	3.44 (0.70)	3.71 (0.92)	3.61 (1.14)	3.44 (0.73)	3.14 (0.86)
Comfort		4.05 (0.91)	3.50 (0.71)	3.65 (0.86)	3.56 (1.15)	3.44 (0.73)	3.29 (0.73)
Ease of care		4.05 (0.71)	3.39 (0.70)	3.65 (1.00)	3.50 (1.15)	3.38 (0.72)	3.00 (0.78)
Overall health		3.95 (0.85)	3.44 (0.70)	3.71 (0.92)	3.83 (0.79)	3.25 (0.77)	3.29 (0.47)

Values are expressed as mean (standard deviation). ROM, Range of Motion; MAS, Modified Ashworth Scale; CPCHILD, Caregiver Priorities and Child Health Index of Life with Disabilities. * *p* value < 0.05.

## Data Availability

Data are contained within the article.
